# Multiscale Simulations Reveal Conserved Patterns of Lipid Interactions with Aquaporins

**DOI:** 10.1016/j.str.2013.03.005

**Published:** 2013-05-07

**Authors:** Phillip J. Stansfeld, Elizabeth E. Jefferys, Mark S.P. Sansom

**Affiliations:** 1 Department of Biochemistry, University of Oxford, South Parks Road, Oxford, OX1 3QU, UK

## Abstract

Interactions of membrane proteins with lipid molecules are central to their stability and function. We have used multiscale molecular dynamics simulations to determine the extent to which interactions with lipids are conserved across the aquaporin (Aqp) family of membrane proteins. Simulation-based assessment of the lipid interactions made by Aqps when embedded within a simple phospholipid bilayer agrees well with the protein-lipid contacts determined by electron diffraction from 2D crystals. Extending this simulation-based analysis to all Aqps of known structure reveals a degree of conservation of such interactions across the Aqp structural proteome. Despite similarities in the binding orientations and interactions of the lipids, there do not appear to be distinct, high-specificity lipid binding sites on the surface of Aqps. Rather Aqps exhibit a more broadly conserved protein/lipid interface, suggestive of interchange between annular and bulk lipids, instead of a fixed annular “shell” of lipids.

## Graphical abstract

**Graphical Abstract:**

**Figure:**
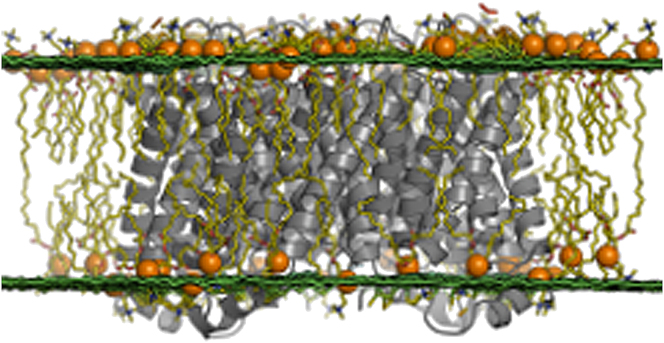

**Highlights:**

► Multiscale MD simulations predict the interactions of lipids with membrane proteins ► The method is evaluated via comparison with the structure of Aqp0 in a membrane ► Simulations of aquaporins reveal a broadly conserved protein/lipid interface ► The results suggest interchange between annular and bulk lipids

## Teaser

Multiscale MD simulations developed by Stansfeld et al. allow prediction of the interactions of lipids with membrane proteins, illustrated here through aquaporins. Results of simulations are in good agreement with available crystal structures and reveal a broadly conserved protein/lipid interface.

## Introduction

Membrane proteins play key roles in a wide range of cellular processes and account for ∼25% of genes. Continued advances in structural biology have resulted in an exponential growth in the number of high-resolution membrane protein structures determined (White, 2009) with ∼2,800 unique structures predicted by 2020. The majority of membrane protein structures determined by X-ray crystallography do not provide direct information on their interactions with their lipid bilayer environment. However, by combining available biophysical and structural data (Killian and von Heijne, 2000; Lee, 2011; Palsdottir and Hunte, 2004), it has been possible to determine some “rules” concerning membrane protein/lipid interactions, revealing, e.g., the importance of tryptophan side chains in positioning a membrane protein relative to the surrounding lipid head groups. There has also been some discussion about the extent to which the structure of a membrane protein may be altered by changes in its immediate lipid and/or detergent environment (Cross et al., 2011; Sanders and Mittendorf, 2011). It is therefore timely to examine the nature of membrane protein interactions with lipid bilayers in detail and to examine to what extent such interactions are conserved across a family of membrane proteins. The Aquaporin (Aqp) family of water permeable pores (Borgnia et al., 1999; Savage et al., 2010) and related proteins provide an opportunity to do this as a number of high-resolution structures have been determined (for a summary, see, e.g., http://blanco.biomol.uci.edu/mpstruc/listAll/list).

A number of high-resolution two-dimensional (2D) crystal structures for Aqp channels in association with phospholipid bilayers have been resolved by electron crystallography (Gonen et al., 2005; Hite et al., 2010; Tani et al., 2009). Two of these are for Aqp0 (Protein Data Bank [PDB] ID codes 2B6O and 3M9I) and a third for Aqp4 (PDB ID code 2ZZ9). 2B6O was resolved in the presence of the lipid dimyristoylphosphatidylcholine (DMPC) (which has two C14:0 acyl tails and a phosphatidyl-choline [PC] head group). 3M9I and 2ZZ9 were resolved in the presence of the *Escherichia coli* polar lipids (EPLs), which were modeled in the structures as diC18:0-phosphatidylethanolamine (DSPE). In the crystal lattices these lipids act as the bridge between crystal neighbors and are thought to reflect the closely interacting annular shell of lipids around the channel in its native lipid bilayer. A recent study has investigated the dynamics of the lipid interactions within the crystal (Aponte-Santamaría et al., 2012).

Including these structures from 2D crystals, there are now approximately 40 high-resolution structures of Aqps. However, for the majority of these structures there is little, if any information, on lipid interactions. Two exceptions are for Aqp1 (2ABM), which contains some fragments of DSPE, and Aqp5 (3D9S), which contains a short-tail phosphatidylserine (PS) lipid. However, in each of these structures the lipid is trapped in the middle of the tetramer, at a site that is unlikely to interact with the surrounding lipid bilayer. For about 20 of the other Aqp structures, bound detergents (either octyl- or nonyl-glucoside detergent) are resolved, sometimes but not always indicating possible bilayer lipid interactions (Figure S1 available online).

Molecular dynamics (MD) simulations of membrane proteins (Stansfeld and Sansom, 2011b) provide an in silico approach to exploring their interactions with lipids in the surrounding bilayer. In particular, coarse-grained (CG) simulations allow one to model assembly and interactions of complex membrane proteins in lipid bilayers (Monticelli et al., 2008). This method has been shown to accurately predict the insertion position of proteins within a cell membrane (Scott et al., 2008). Previous studies enabled us to correlate predicted contacts to lipids with experimental data on lipid-exposed residues for a number of membrane proteins (e.g., LacY, rhodopsin, KcsA, MscL, FepA, and BtuB). Furthermore, a prediction of the PIP_2_ binding site in Kir channels has been validated through high-resolution X-ray crystallography structures (Hansen et al., 2011; Stansfeld et al., 2009). The ability to convert coordinate sets from CG simulations to atomistic resolution permits direct comparison between the atomistic simulations and electron microscopy (EM) structures of the Aqp channels, while also allowing more detailed contact information (e.g., hydrogen bonds) between lipids and proteins to be derived. Through comparison with the atomistic simulations it is also possible to validate the CG methodology as a suitable initial approximation for describing lipid-protein interactions.

Molecular simulations of Aqps (de Groot and Grubmüller, 2001; Jensen et al., 2001; Savage et al., 2010; Tajkhorshid et al., 2002) and related computational studies (Oliva et al., 2010) have been especially successful in enabling dissection of the mechanism of selective water and/or solute permeation of through the central pore of the protein. Here, we employ a multiscale MD simulation approach to characterize the interactions of Aqps with their lipid bilayer environment, revealing the degree of conservation of such interactions across the Aqp structural proteome and thus demonstrating the likely functional/structural importance of such conserved interactions.

## Results and Discussion

### Comparison of Lipid Contacts: Simulations versus Crystal Structures

Our overall aim in this study is to investigate lipid/protein interactions across a family of membrane proteins for which structures are available. This is anticipated to reveal conserved patterns of lipid interaction and thus add to the information available directly from the crystal structure. Aqps that were selected as a number of high-resolution structures (Aqp0: PDB ID codes 2B6O and 3M9I; Aqp-4: PDB ID code 2ZZ9) and were derived from two-dimensional (2D) electron crystallography have recently emerged. These structures have revealed the location of bilayer lipids, thus allowing us to validate our simulation-based predictions against experimental structural data.

**Figure 1:**
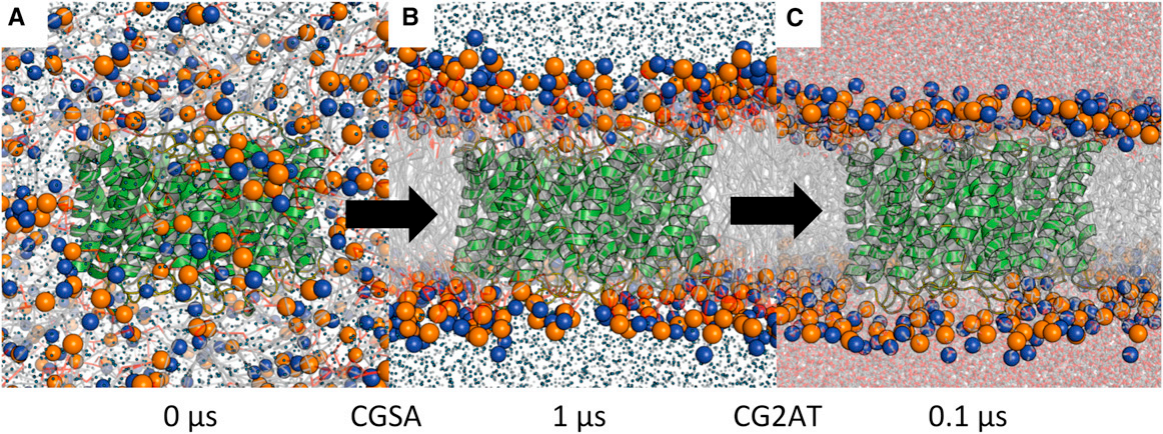
Multiscale Simulations of Aqp0 (A) The Aqp0 protein tetramer, as a coarse-grained (CG) model, is positioned within a simulation box of randomly placed lipids (head groups shown as orange and blue spheres for phosphate and choline groups, respectively). (B) After a 1 μs CG-MD simulation, the bilayer has formed around the protein (CGSA), and the protein/lipid interactions have reached equilibrium. (C) The CG system is converted to atomistic (AT) representation (CG2AT), and a subsequent 0.1 μs MD simulation is performed. See also Movie S1.

Our multiscale simulation protocol, as described previously (Stansfeld and Sansom, 2011a), combines a coarse-grained molecular dynamics (CG-MD) simulation, in which a lipid bilayer is self-assembled around the experimentally determined protein structure, conversion of the resultant protein/membrane system from coarse-grained to atomistic representation (CG2AT), and subsequent atomistic molecular dynamics (AT-MD) simulation of the protein embedded in a lipid bilayer (see Figure 1). The CG-MD simulation allows the bilayer lipids to equilibrate around the protein, whereas the subsequent AT-MD simulations allow refinement and more detailed analysis of lipid-protein interactions.

In addition to the bilayer self-assembly simulations, we also performed multiscale simulations of the Aqp-plus-lipid tetrameric and octameric units (as present in the double-layered 2D crystals), to permit a more direct comparison between simulations and the 2D crystals. All atomistic simulations showed robust stability of the Aqp structures with root-mean-square deviations in the region of 3 Å or below, with simulations of the relatively tightly packed EM crystal plateauing at ∼2 Å.

For each simulation of the Aqp EM structures, we used the lipid that was present in the crystals. In two of the crystals (3M9I and 2ZZ9), the lipid present is DSPE, whereas in 2B6O, it is DMPC. For the three crystal structures, the total number of lipids forming the annulus around the protein structure is 72 for 2B6O, 56 for 3M9I, and 40 for 2ZZ9. From the CG-MD bilayer self-assembly simulations, the average number of interacting lipids (defined as those with their phosphate particle within 6 Å of the protein; a CG interparticle cutoff of 6 Å has been shown to be comparable to an interatomic cutoff of 4 Å [Stansfeld and Sansom, 2011a]) is 81 for 2B6O, 64 for 3M9I, and 63 for 2ZZ9. The agreement between 3M9I and 2ZZ9 for the same lipid type suggests that the reduced number of lipids interpreted as present in the EM structure of 2ZZ9 may reflect the reduced lipid density rather than a smaller annular shell around Aqp4.

**Figure 2:**
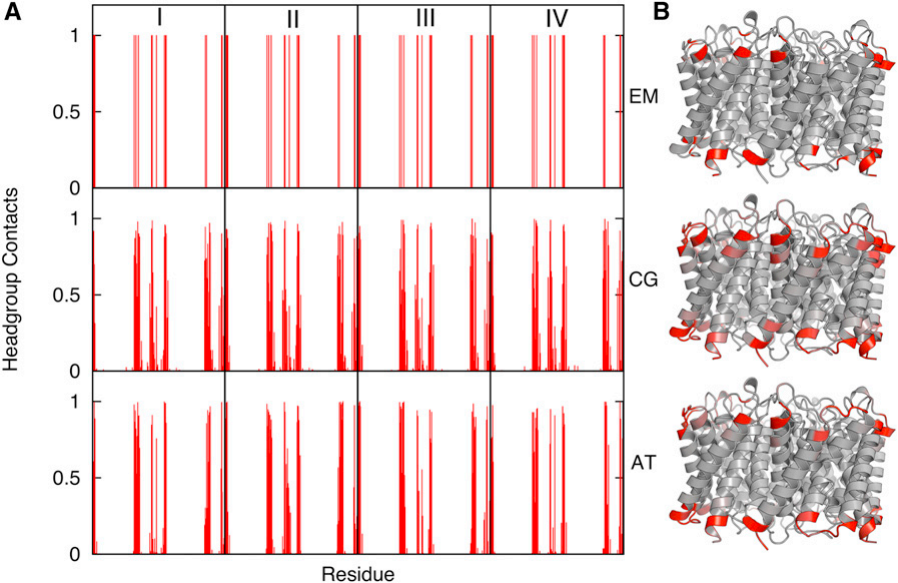
Comparison between Experimental and Simulation Lipid-Residue Interactions (A) Protein-lipid head group contacts in the two-dimensional crystal of Apq0 (PDB ID code 3M9I) compared with the interactions during the CG- and AT-MD simulations for all four Aqp subunits (I-IV). The residues interacting with the bound lipids in the atomistic structures are calculated based on a 4 Å cutoff, with 6 Å used for the CG simulations. (B) The contacts are colored onto their corresponding residues using a white (low) to red (high) gradient. See also Figure S2.

We may compare the protein contacts to these lipids on a residue-by-residue basis (Figure 2). For all three structures, there is a good correlation, for both the interactions of the head group and for the alkyl tails between the residues identified in the simulations and those in the crystal structures (Table 1). Notably, almost all of the interactions observed in the crystal structures are also detected in the simulations, with the vast majority forming contacts that persist throughout the simulations. Although it is unsurprising that the outer surface of the protein forms the majority of the contacts with the lipid, the exact nature and residue identities of the interactions would not be obvious simply via examination of the protein crystal structures in the absence of lipids. Thus, we have demonstrated the ability of our simulation protocol to add back the information missing from the great majority of Aqp crystal structures, testing this approach using those structures in which a lipid bilayer is present in the crystal.

It is important to note that the majority of the predicted interactions are already identified in the CG-MD simulations, reinforcing earlier suggestions that such simulations are able to predict experimentally observed protein/lipid contacts (Scott et al., 2008). Some (∼15%) additional interactions are observed in the simulations that were not observed in the crystal structures. The overall correlation coefficients between the protein lipid contacts seen in the self-assembly simulations and those seen in the 2D crystal structures are approximately 0.6 to 0.7 (see Table 1). The correlation coefficients when comparing contacts in the EM structures are ∼0.62 for both the head groups and for the alkyl tail interactions.

It seems likely that the additional contacts seen in the simulations may reflect (1) the difference between the 2D crystal environment and a bilayer environment and (2) the enhanced mobility of protein and lipids in the simulations at near room temperatures. We therefore also compared the contacts present in simulations of Aqp0 in a self-assembled bilayer with the contacts present in simulations starting from the 2D crystal structures containing both proteins and lipids. Thus, we compared predicted interactions with “thermalized” lipids from the crystal structures. This resulted in an increase in the correlation coefficients between ∼0.8 (for 2B6O) and 0.9 (for 3M9I; Table 1). This is true whether one considers contacts formed by the head groups or the alkyl tails. This suggests that once one allows for room temperature dynamics of the crystal structure, the self-assembly simulation protocol achieves a high accuracy in predicting the experimentally observed protein/lipid contacts.

The smaller number of lipid observed in the Aqp4 2ZZ9 (Table 1) structure results in lower correlation coefficients (0.54 for the head groups and 0.47 for the tails) for the EM structure compared to the CG simulations. Unfortunately, in this case it was also not possible to simulate lipid-protein interactions starting directly from the EM structure as a number of the lipids in the structure were missing atoms within their acyl tails. This therefore excluded calculation of correlations involving a simulated EM structure.

Previous comparison of the lipid contacts in the two crystal structures of Aqp0 has been interpreted as indicating that the interactions of the annular lipids are driven by packing of the acyl chains to the protein surface rather than specific protein/head group interactions (Hite et al., 2010). We therefore performed self-assembly simulations of the interactions of Aqp0 with a third phospholipid, namely, dipalmitoylphosphatidylcholine (DPPC) (diC16:0-PC; as used in previous simulation studies of membrane proteins; Stansfeld and Sansom, 2011a). We observed strong correlation between the contacts seen in AT-MD simulations of the 2D crystals and in CG-MD simulations in self-assembled DPPC bilayers. Thus, for 2B6O, the correlation coefficients were 0.76 for head groups and 0.80 for the tails; for 3M9I, they were 0.88 for head groups and 0.82 for the tails. This suggests that the contact residues identified in simulations (and in the 2D crystal structures) are robust to small changes in lipids (we note that all three lipids are zwitterionic), and so DPPC was used in subsequent simulations across the whole family of Aqp structures.

**Figure 3:**
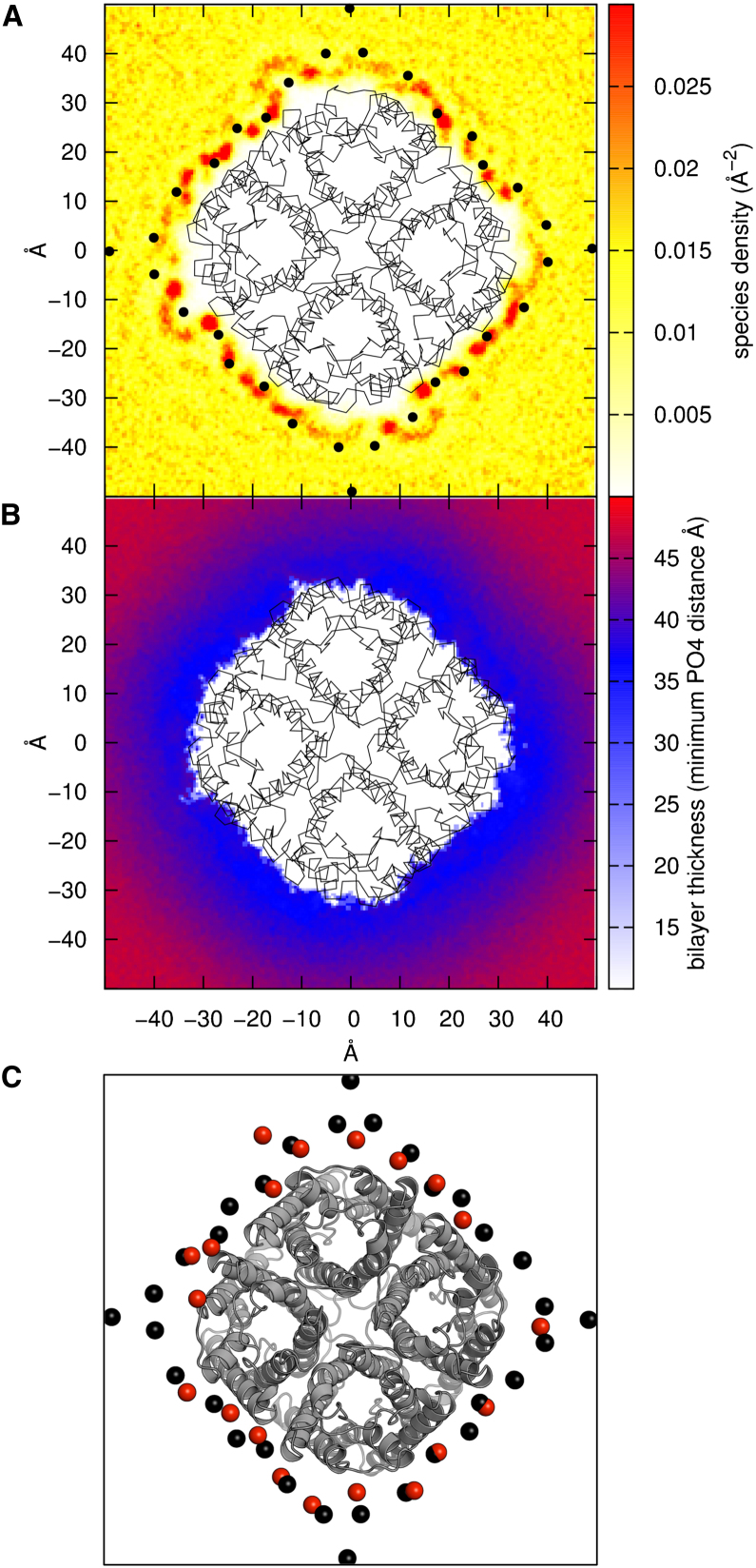
Lipid Density and Thickness (A) Lipid (phosphate particle) probability density in the extracellular leaflet around Aqp0 (3M9I) from the CG self-assembly simulation. The density is compared with the positions of the lipids resolved in the two-dimensional crystal, for which the phosphorus atoms of the resolved lipids are shown as black spheres. (B) The thickness of the lipid bilayer around Aqp0, from the simulation of 3M9I with DSPE. The DSPE bilayer can be seen to thin substantially in the vicinity of the protein, from ∼50 to 35 Å. (C) A snapshot of the 3M9I Aqp0 CG simulation at 1 μs compared with phosphate particles within 6 Å shown in red spheres. The phosphate atoms captured in the EM structure are shown in black spheres. See also Figure S3.

One may also compare the spatial distributions of lipids in the simulations and in the 2D crystals. From the simulations it is evident that there is a first shell or annulus (Lee, 2011) of spatially immobilized lipids. This is most marked in the outer leaflet of the bilayer, where there appear to be a number of clearly defined lipid binding sites. If one examines the peaks of phosphate density in the bilayer plane for the simulations (Figure 3A), these correlate well between, e.g., the Aqp0/DMPC and Aqp0/DSPE simulations. They also correlate reasonably well with the positions of the lipid phosphates in the two Aqp0 crystal structures. That the correlation with the experimental structures is not perfect can be understood both in terms of the more dynamic localization of lipids in the simulations and also given the conclusion of Hite et al. (2010) that acyl chain packing, rather than head group interactions, are better conserved between the two crystal structures, a conclusion that was supported by MD simulations of the EM crystal structures (Aponte-Santamaría et al., 2012).

**Figure 4:**
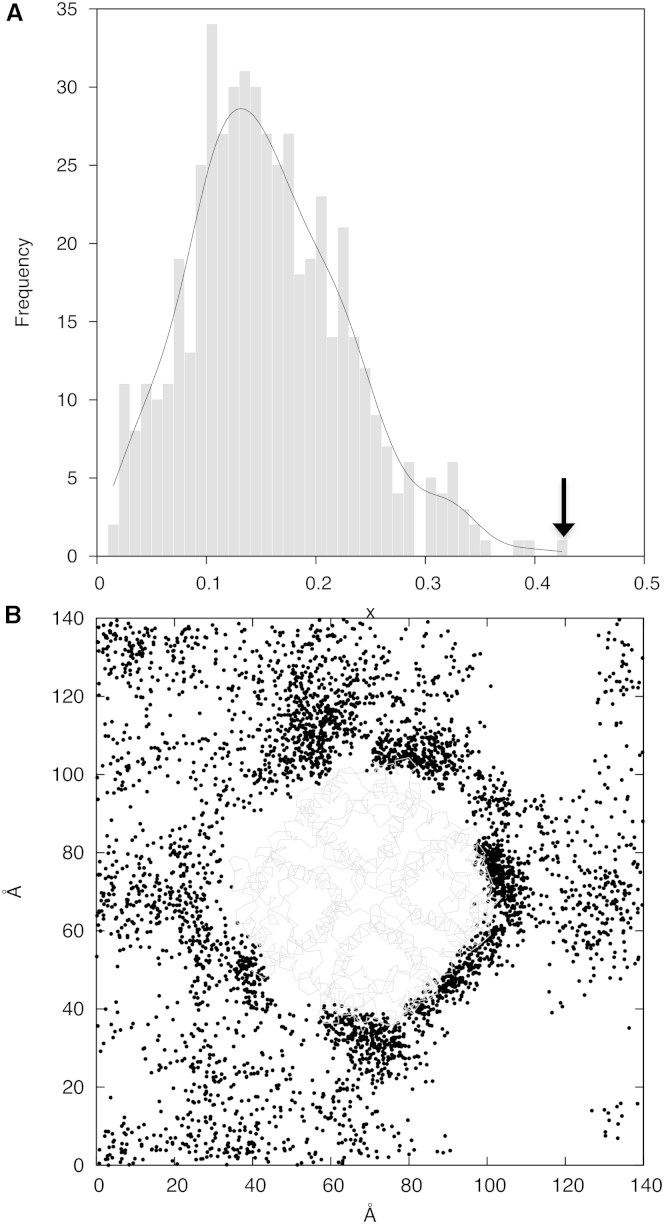
Lipid-Protein Interactions Are Short Lived (A) Distribution of fractional protein contact times for individual DMPC lipids during 1μs CG-MD simulation of the 2B6O Aqp0 structure. A residue that interacts with a lipid throughout the simulation has a value of one. (B) Phosphate particle positions, shown for every frame within the bilayer plane, for a lipid that had the most protein contacts over the course of the simulation, indicated by the arrow in (A). The backbone trace of the central Aqp0 tetramer is shown in gray.

Although there appear to be preferential lipid binding sites in the simulations, the lipids do not seem to remain bound to the protein for the entirety of the CG simulations, but rather to exchange with the surrounding “bulk” lipids. Thus, we have examined the distribution of times for which a given lipid molecule contacts the Aqp0 surface over the duration of a 1 μs CG-MD simulation (Figure 4). From this it is evident that there is a major population contacting the lipid for ∼15% of the simulation but also a minor population that contacts the lipid for approximately 30% to 40% of the simulation. It is this population that constitutes the annulus of partially immobilized lipids discussed above. However, if we examine even the most “tightly bound” lipid from this distribution (which is in contact with Aqp0 for >40% of the 1 μs simulation; Figure 4B), we can see that while residing at distinct interaction sites on the protein surface, this lipid also exchanges with those in the surrounding bilayer.

Comparison of the crystal structures of Aqp0 revealed that despite the differences in acyl tail length, the distance between the phosphate atoms in the upper and lower leaflets are the same, with a phosphate-phosphate distance of ∼35 Å (Hite et al., 2010). This agrees well with the average phosphate-phosphate distance observed in a simulated DMPC bilayer. This lipid appears to be well suited to accommodate the Aqp0 structures; the “bulk” and “annular” lipid thickness are the same (Figure S3). In contrast, for “bulk” DSPE, this distance is ∼50 Å, but this is reduced locally to ∼35 Å in the annular regions, thus overcoming the hydrophobic mismatch (Figure 3). In the DPPC bilayers, the thickness in the bulk region is ∼40 Å, falling locally to ∼35 Å in the annulus. Thus, for Aqp0, the hydrophobic thickness of the protein dictates the local thickness of the bilayer. This has been suggested by previous theoretical (Mouritsen and Bloom, 1984) and model system (Killian, 1998) studies as one of a range of possible responses to protein/bilayer thickness mismatch. This also helps to explain why Aqps are more structurally and functionally tolerant to changes in membrane lipid composition (Sanders and Mittendorf, 2011) than are more conformationally labile small membrane proteins, such as influenza M2 (Cross et al., 2011).

The phosphate-phosphate distance of 35 Å, regardless of changes in lipid head group and/or tail length, suggests that Aqps are likely to reside in relatively thin domains within the membrane. This may help to explain why β-octyl glucoside, a comparatively short alkyl chain detergent, has been successfully applied as a membrane mimetic in crystallization of a number of Aqps, with 19 structures captured with density resolved for the detergent molecules (Figure S1). Preliminary simulation studies of protein-detergent interactions reveal the nature of a “bilayer-like micelle” formed by β-octyl glucoside around GlpF (Figure S5). Further investigations into the interactions of different detergents with membrane proteins may enable rationalization of the optimal detergents for crystallization of different membrane proteins.

### Multiscale Simulations of Lipid Interactions across the Aqp Family

**Figure 5:**
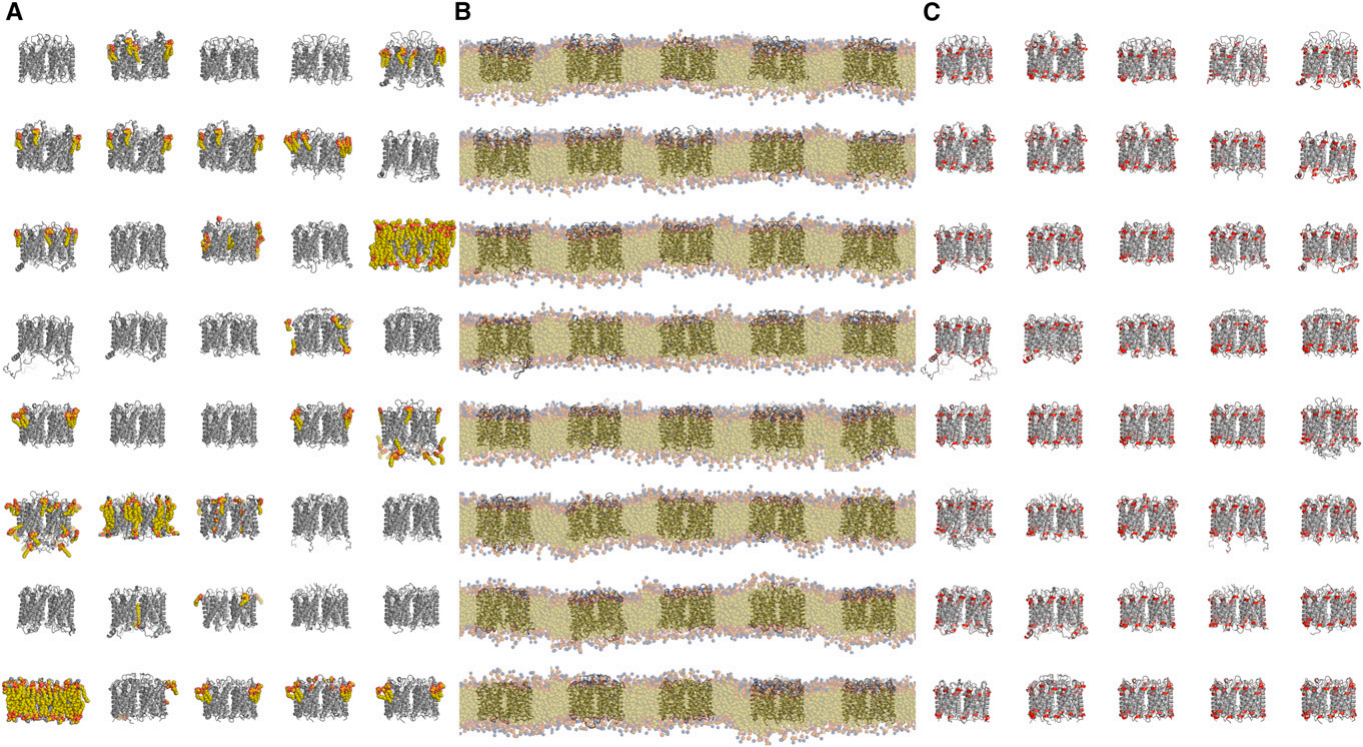
Simulations of the Aqp Family (A) The 40 structures of Aqps and aquaglyceroporins in the PDB. (B) CG-MD simulations in which phospholipid (DPPC) bilayers were self-assembled around the protein structures. (C) The predicted lipid head group/protein contacts analyzed and displayed. This corresponds to a total of 40 × 1 μs of CG-MD simulations. See also Figures S1 and S5.

From these simulation studies, and from the experimental structural data, an apparent paradox emerges concerning the nature of the interactions of Aqp0 with lipid head groups. It is evident that although the phospholipids do not occupy highly (head group) specific “tight binding” sites, the P-P distance is, however, robust to changes in exact lipid head group species and in acyl tail length. This suggests a degree of structural/functional importance of lipid interactions with the protein that one might expect to be reflected in a degree of evolutionary conservation of these interactions. So, in order to see if similar interactions are observed across the Aqp family, the CG self-assembly approach was repeated for all 40 Aqp structures in the PDB, using DPPC as a simple zwitterionic lipid. For each Aqp system, a lipid bilayer self-assembled within 100 ns of CG simulation (Figure 5). The simulations were then extended to 1 μs before analysis of protein/lipid interactions. Subsequent atomistic simulations were also performed for 20 ns per channel structure.

**Figure 6:**
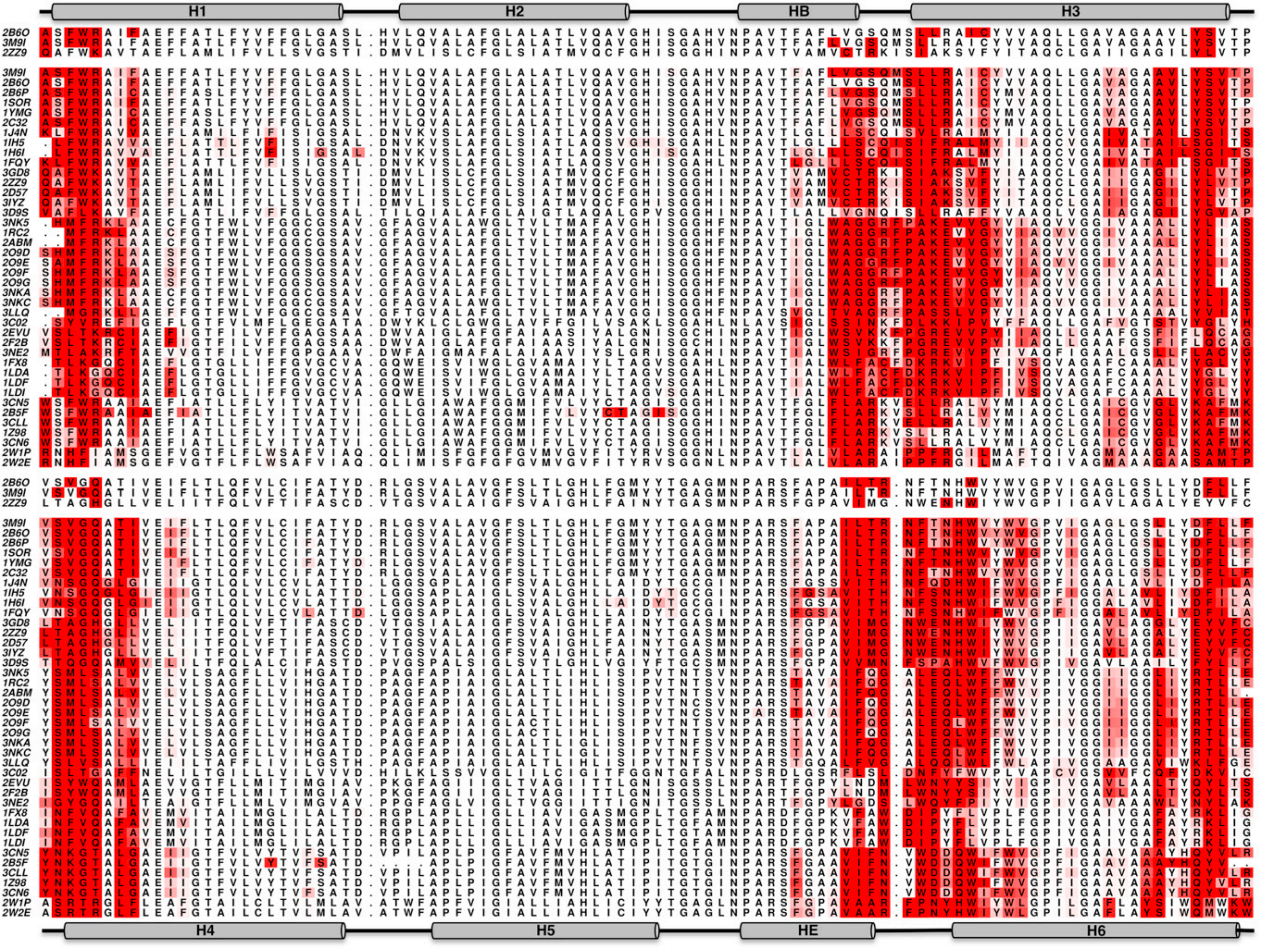
Sequence Alignment Showing Aqp-Lipid Head Group Interactions All contacts within 6 Å from the CG simulations of 40 different Aqp (shown as a white to red gradient) are displaced on a corresponding sequence alignment. The upper half of the alignment shows TM helices H1 to H3, and the lower half shows the symmetry-related repeat in TM helices H4 to H6. The first three lines of the alignment correspond to 2B6O, 3M9I, and 2ZZ9 (with a cutoff of 4 Å), and the contacts seen in the crystal structures are colored red. See also Figure S4.

The degree of conservation of the interactions may be viewed in terms of an alignment of the Aqp sequences, with residues colored on the frequency of lipid head group (Figure 6) or alkyl tail (Figure S4) interactions. From analysis of the lipid head group interactions, it is evident that there are two regions “locking” the protein into position in the bilayer, one intracellular (IC) and one extracellular (EC), which are related to one another by the known 2-fold pseudosymmetry of the Aqp fold. Thus, the IC “lock” is formed by the N terminus of transmembrane helix H1, the C terminus of the short HB helix, the N terminus of H3, and the C terminus of H6. This pattern of interactions is related by the pseudo-2-fold symmetry to the EC “lock” made up of the N terminus of transmembrane helix H4, the C terminus of the short HE helix, the N terminus of H6, and the C terminus of H3. All of these regions show a degree of conservation of amino acid composition, with numerous basic (K,R) and aromatic (W,Y) side chains present, but do not show highly conserved sequence motif. Of course, it is well known that these residues are found with high frequency at the termini of transmembrane α helices and play a key role in “locking” these helices into place relative to a lipid bilayer (Killian and von Heijne, 2000). Thus, our results indicate conservation of the structural feature of positioning of the Aqp in a bilayer rather of specific tight binding sites for lipids.

### Residue Types Involved in Lipid Interactions

**Figure 7:**
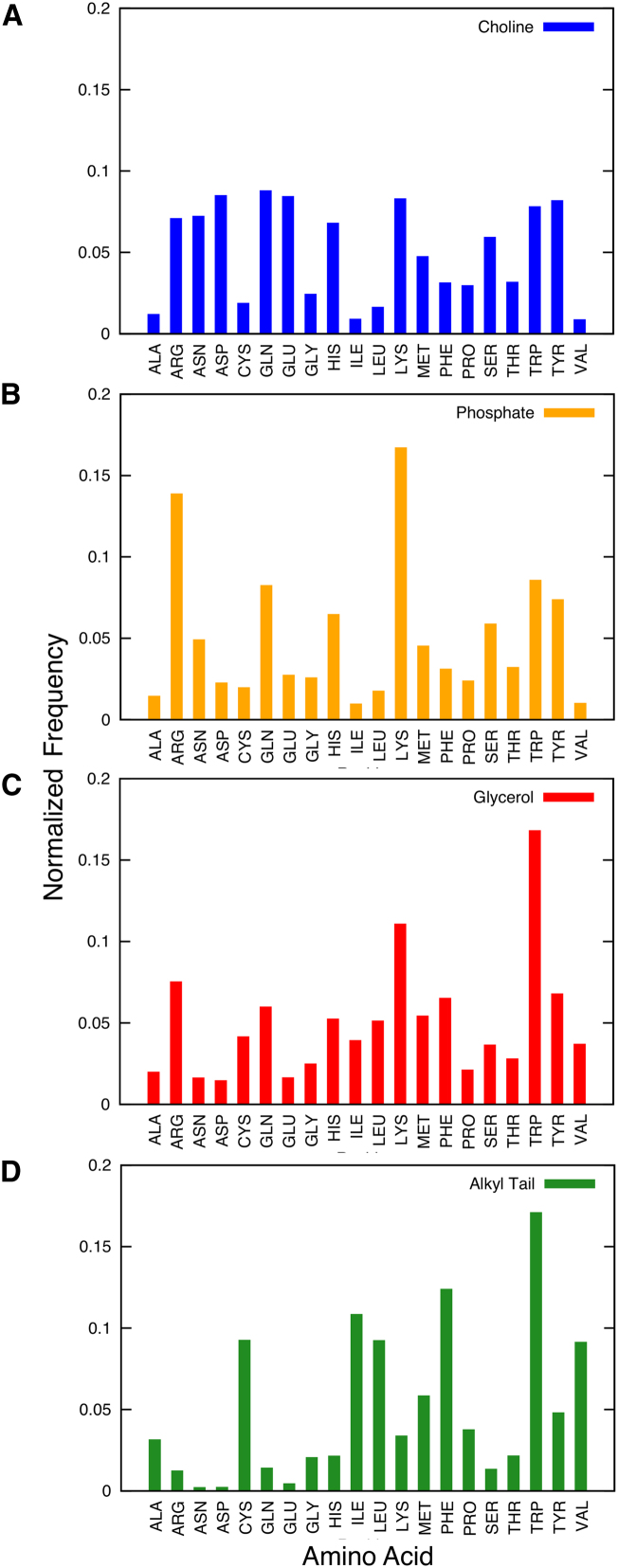
Specific Lipid-Residue Interactions (A–D) Percentage of specific residue interactions with segments of the lipids as observed in the 1 μs CG simulations of the 40 aquaporin structures. (A) Choline (blue). (B) Phosphate (orange). (C) Glycerol (red). (D) Tail (green). Each residue type is normalized against its relative occurrence within the 40 protein structures.

From the data set of 40 simulations of Aqp/lipid interactions, it is possible to extract the relative frequency of the different amino acid types that interact with different regions of the lipid molecules, thus providing an Aqp-centric view of the surrounding lipid environment (Figure 7). Thus, the choline group is found in close proximity with aspartate, asparagine, glutamate, and glutamine. In contrast, for the lipid phosphate groups, the predominant contacts are with arginine and lysine side chains, reflecting local electrostatic interactions. For the glycerol region, tryptophan residues form many of the contacts in agreement with the known role of tryptophan side chains in “locking” membrane proteins in a lipid bilayer (Norman and Nymeyer, 2006) and the preferred location of indole groups within model lipid bilayers (Norman and Nymeyer, 2006; Yau et al., 1998). Tryptophan residues also interact with the lipid tails, along with phenylalanine and other hydrophobic residues.

**Figure 8:**
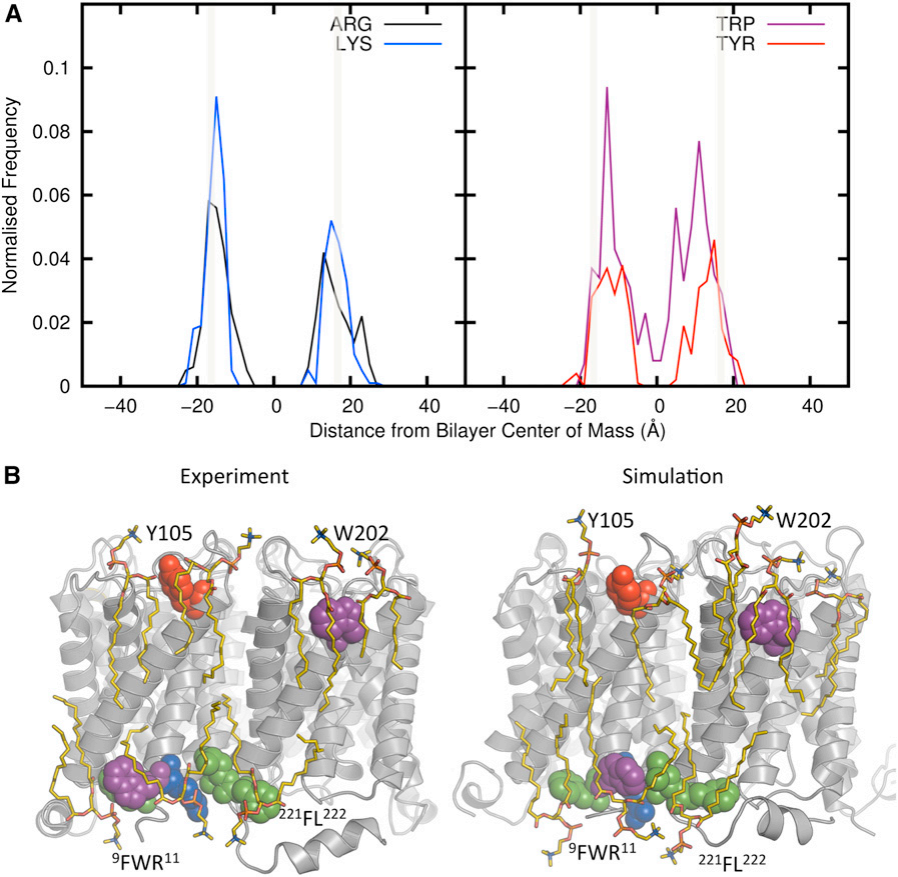
Residue Distribution and Lipid Binding Sites (A) The distributions of Trp, Tyr, Arg, and Lys across the bilayer (along the z axis). The head group regions of the bilayer are marked by gray bars. As expected, these residues principally interact with the membrane within the head group region. (B) Comparison of lipid-protein contacts in Aqp0. The position of the lipids (yellow, bond format) and the protein (gray ribbons) are compared for the crystal structure and after 100 ns of AT-MD simulation of the 3M9I structure. Side chains that form contacts with lipid head groups in experimental and the simulated structures are shown in spheres. These include the relatively well conserved ^9^FWR^11^ motif at the N-terminal end of H1, Y105 in H3, and W202 and ^221^FL^222^ in H6. See also Movie S1.

The amino acid residues that form the interactions “locking” Aqps into their correct location in the bilayer may be viewed in terms of the distribution of the key amino acid residue types onto the bilayer normal (Figure 8A). From these distributions, it can be seen that the two types of residue noted above (aromatic and basic interactions with the head group) clearly correspond to two bands of interactions with the lipid head groups. Thus, in this respect, the Aqps provide a clear example of the pattern of membrane protein/lipid head group interaction previously suggested on the basis of analyses of known structures (e.g., Lee, 2003; Palsdottir and Hunte, 2004; Ulmschneider and Sansom, 2001).

In this context, we may return to the multiscale simulations of Aqp0 in a bilayer and examine the detailed interactions presented in a snapshot from the end of an AT-MD simulation in DMPC (Figure 8B). From this it is evident that not only does the simulation well reproduce the interactions seen in the 2D crystal but also that it provides a dynamic view (Movie S1) of the protein/head group interactions “locking” the protein into a phospholipid bilayer. These include the relatively well-conserved F^9^WR^11^ motif at the N terminus of transmembrane helix H1, Y105 of helix H3, and W202 of helix H6.

### Conclusions

In this study our simulation-based prediction of the lipid interactions made by Aqps when embedded within a simple phospholipid bilayer agrees favorably with the protein/lipid contacts determined by electron diffraction from 2D crystals. This validation provided us with the confidence to extend the simulation-based analysis to all Aqps of known structure. The analysis of 40 such simulations suggests that despite similarities in the binding orientations and interactions of the lipids, there do not appear to be distinct, high-specificity lipid binding sites on the surface of Aqps (Sanders and Mittendorf, 2011). Rather, there is a more broadly conserved protein/lipid interface, enabling dynamic interplay between the lipid and protein. This differs from (tighter) binding of specific lipids to sites on the protein surface, as seen in, e.g., potassium channels (KcsA [Williamson et al., 2002] and Kir/PIP_2_ [Hansen et al., 2011; Stansfeld et al., 2009]), and also in a number of mitochondrial and related proteins interacting with cardiolipins (Palsdottir and Hunte, 2004). The results for the Aqps are suggestive of a model of interchange between annular and bulk lipids, rather than a fixed annular “shell” of lipids. This latter conclusion is supported by observation of dynamic binding/unbinding of lipids during the course of 1 μs CG-MD simulations. Thus, multiscale simulations are able to extend our view of membrane proteins as obtained from crystallographic structure determination, providing details of the interactions of membrane proteins with their lipid bilayer environment. In particular, the CG simulations are able to adequately capture the overall pattern of protein/lipid in interactions, as exemplified by two recent studies of cytochrome /cardioliopin interactions (Arnarez et al., 2013a, 2013b), whereas atomistic simulations enable the details of, e.g., lipid head-group-protein H-bonds to be explored in more detail, as seen in a recent study of Kir channel/PIP_2_ interactions (Schmidt et al., 2013). So, in light of the functional importance of many lipid/protein interactions (Lee, 2004), this provides a significant addition to our toolkit for studying the structural biology of membrane proteins. It would therefore be timely to apply the analysis described in this paper to other families of membrane proteins. Aquaporins seems to be especially functionally resilient to changes in their lipid environment (Sanders and Mittendorf, 2011). However, this may not be the case for all membrane proteins, and so a wider range of understanding of protein/lipid interactions is needed.

## Experimental Procedures

All Aqp structures were downloaded in their tetrameric form from the PDB (Berman et al., 2003). The biological unit of 3M9I was modified so that the lipids were on the outer face of the tetramer. In the case of 3IYZ (10 Å resolution), Pulchra (Rotkiewicz and Skolnick, 2008) was used to add the missing atoms to the Cα-trace. All structures were subsequently parsed through PDB2PQR (Dolinsky et al., 2004) to insert any other missing atoms. The missing lipid atoms, in the 2D crystal structures, were added by appending minimized fragments of the acyl tails using PyMOL (DeLano, 2002).

### Multiscale Simulations in Self-Assembled Lipid Bilayers

Simulations were run using Gromacs (v. 4.5.3) (Hess et al., 2008). Self-assembly CG simulations were performed for 1 μs at 323 K using a modified version (Bond et al., 2008) of the MARTINI (Marrink et al., 2007; Monticelli et al., 2008) force field. For 2B6O, 3M9I, and 2ZZ9 structures, the same lipid type was used as in the corresponding 2D crystal, namely, DMPC and DSPE. The final systems were then converted to atomistic detail using the CG2AT protocol (Stansfeld and Sansom, 2011a) and run for a further 100 ns of AT-MD with the Gromos96 43a2 force field (van Gunsteren et al., 1996). Furthermore, 40 Aqp structures were subjected to 1 μs CG self-assembly simulations in the presence of DPPC lipid and then run for 20 ns of AT-MD after conversion (Table 1). For all CG simulations, any lipids outside the bilayer were removed at 100 ns, once the bilayer had completely formed. All analyses were performed between 200 ns and 1 μs of CG simulation. Pearson’s *r* was used for the correlation coefficient analysis.

### Multiscale Simulations of 2D Crystals

Because of the incomplete determination of the lipids in the 2ZZ9 structure, simulations of the crystal complexes were only performed for 2B6O and 3M9I. The protein structures were converted to CG as described previously. Lipid atoms were mapped to their CG counterparts. Simulations were run for 1 μs. Atomistic simulations of the protein-lipid complexes from the crystal structures were performed for 100 ns. CG and AT simulations were performed for the octameric junctional complex in a double bilayer system.

## Figures and Tables

**Table 1:** Correlation Matrix of Lipid Contacts

Head Group							
Tails	2B60	EM	AT (SA)	AT (EM)	CG (SA)	CG (EM)	DPPC
	EM		0.56	0.56	0.62	0.64	0.64
	AT (SA)	0.65		**0.84**	0.81	0.75	0.84
	AT (EM)	0.75	**0.83**		0.76	0.70	0.76
	CG (SA)	0.70	0.83	0.80		0.96	0.97
	CG (EM)	0.71	0.76	0.77	0.96		0.94
	DPPC	0.70	0.82	0.80	0.98	0.95	

**Head Group**							

Tails	3M9I	EM	AT (SA)	AT (EM)	CG (SA)	CG (EM)	DPPC
	EM		0.62	0.63	0.57	0.54	0.56
	AT (SA)	0.62		**0.91**	0.90	0.82	0.88
	AT (EM)	0.66	**0.93**		0.89	0.85	0.88
	CG (SA)	0.66	0.82	0.83		0.93	0.99
	CG (EM)	0.67	0.80	0.82	0.97		0.93
	DPPC	0.67	0.81	0.82	1.00	0.98	

**Head Group**							

Tails	2ZZ9	EM	AT (SA)		CG (SA)		DPPC
	EM		0.30		0.47		0.45
	AT (SA)	0.45			0.71		0.75
	CG (SA)	0.54	0.83				0.95
	DPPC	0.47	0.78		0.90		

Correlation of contacts for the two AQP0 structures (2B60 and 3M9I) and the Aqp4 structure (2ZZ9), comparing the interactions observed in the EM structure with the CG self-assembly (SA) simulations and (in the case of the two Aqp0 simulations) those using the EM structure as the starting point. A comparison with CGSA simulations using DPPC, rather than DSPE or DMPC, is also shown. The correlation coefficients between the atomistic simulations of the EM structure and following CGSA are in bold.

## Supplemental Information

Document S1. Figures S1–S5 and Table S1

Movie S1. Lipid Interactions with Aqp0, Related to Figures 1 and 8

## References

[bib1] Aponte-Santamaría C., Briones R., Schenk A.D., Walz T., de Groot B.L. (2012). Molecular driving forces defining lipid positions around aquaporin-0. Proc. Natl. Acad. Sci. USA.

[bib2] Arnarez C., Marrink S.J., Periole X. (2013). Identification of cardiolipin binding sites on cytochrome c oxidase at the entrance of proton channels. Sci. Rep..

[bib3] Arnarez C., Mazat J.-P., Elezgaray J., Marrink S.-J., Periole X. (2013). Evidence for cardiolipin binding sites on the membrane-exposed surface of the cytochrome bc1. J. Am. Chem. Soc..

[bib4] Berman H., Henrick K., Nakamura H. (2003). Announcing the worldwide Protein Data Bank. Nat. Struct. Biol..

[bib5] Bond P.J., Wee C.L., Sansom M.S.P. (2008). Coarse-grained molecular dynamics simulations of the energetics of helix insertion into a lipid bilayer. Biochemistry.

[bib6] Borgnia M., Nielsen S., Engel A., Agre P. (1999). Cellular and molecular biology of the aquaporin water channels. Annu. Rev. Biochem..

[bib7] Cross T.A., Sharma M., Yi M., Zhou H.X. (2011). Influence of solubilizing environments on membrane protein structures. Trends Biochem. Sci..

[bib8] de Groot B.L., Grubmüller H. (2001). Water permeation across biological membranes: mechanism and dynamics of aquaporin-1 and GlpF. Science.

[bib9] DeLano, W.L. (2002). The PyMOL molecular graphics system. http://www.pymol.org.

[bib10] Dolinsky T.J., Nielsen J.E., McCammon J.A., Baker N.A. (2004). PDB2PQR: an automated pipeline for the setup of Poisson-Boltzmann electrostatics calculations. Nucleic Acids Res..

[bib11] Gonen T., Cheng Y., Sliz P., Hiroaki Y., Fujiyoshi Y., Harrison S.C., Walz T. (2005). Lipid-protein interactions in double-layered two-dimensional AQP0 crystals. Nature.

[bib12] Hansen S.B., Tao X., MacKinnon R. (2011). Structural basis of PIP_2_ activation of the classical inward rectifier K^+^ channel Kir2.2. Nature.

[bib13] Hess B., Kutzner C., van der Spoel D., Lindahl E. (2008). GROMACS 4: algorithms for highly efficient, load-balanced, and scalable molecular simulation. J. Chem. Theory Comput..

[bib14] Hite R.K., Li Z., Walz T. (2010). Principles of membrane protein interactions with annular lipids deduced from aquaporin-0 2D crystals. EMBO J..

[bib15] Jensen M.O., Tajkhorshid E., Schulten K. (2001). The mechanism of glycerol conduction in aquaglyceroporins. Structure.

[bib16] Killian J.A. (1998). Hydrophobic mismatch between proteins and lipids in membranes. Biochim. Biophys. Acta.

[bib17] Killian J.A., von Heijne G. (2000). How proteins adapt to a membrane-water interface. Trends Biochem. Sci..

[bib18] Lee A.G. (2003). Lipid-protein interactions in biological membranes: a structural perspective. Biochim. Biophys. Acta.

[bib19] Lee A.G. (2004). How lipids affect the activities of integral membrane proteins. Biochim. Biophys. Acta.

[bib20] Lee A.G. (2011). Biological membranes: the importance of molecular detail. Trends Biochem. Sci..

[bib21] Marrink S.J., Risselada H.J., Yefimov S., Tieleman D.P., de Vries A.H. (2007). The MARTINI force field: coarse grained model for biomolecular simulations. J. Phys. Chem. B.

[bib22] Monticelli L., Kandasamy S.K., Periole X., Larson R.G., Tieleman D.P., Marrink S.J. (2008). The MARTINI coarse grained force field: extension to proteins. J. Chem. Theory Comput..

[bib23] Mouritsen O.G., Bloom M. (1984). Mattress model of lipid-protein interactions in membranes. Biophys. J..

[bib24] Norman K.E., Nymeyer H. (2006). Indole localization in lipid membranes revealed by molecular simulation. Biophys. J..

[bib25] Oliva R., Calamita G., Thornton J.M., Pellegrini-Calace M. (2010). Electrostatics of aquaporin and aquaglyceroporin channels correlates with their transport selectivity. Proc. Natl. Acad. Sci. USA.

[bib26] Palsdottir H., Hunte C. (2004). Lipids in membrane protein structures. Biochim. Biophys. Acta.

[bib27] Rotkiewicz P., Skolnick J. (2008). Fast procedure for reconstruction of full-atom protein models from reduced representations. J. Comput. Chem..

[bib28] Sanders C.R., Mittendorf K.F. (2011). Tolerance to changes in membrane lipid composition as a selected trait of membrane proteins. Biochemistry.

[bib29] Savage D.F., O’Connell J.D., Miercke L.J., Finer-Moore J., Stroud R.M. (2010). Structural context shapes the aquaporin selectivity filter. Proc. Natl. Acad. Sci. USA.

[bib30] Schmidt M.R., Stansfeld P.J., Tucker S.J., Sansom M.S.P. (2013). Simulation-based prediction of phosphatidylinositol 4,5-bisphosphate binding to an ion channel. Biochemistry.

[bib31] Scott K.A., Bond P.J., Ivetac A., Chetwynd A.P., Khalid S., Sansom M.S.P. (2008). Coarse-grained MD simulations of membrane protein-bilayer self-assembly. Structure.

[bib32] Stansfeld P.J., Sansom M.S.P. (2011). From coarse-grained to atomistic: a serial multi-scale approach to membrane protein simulations. J. Chem. Theory Comput..

[bib33] Stansfeld P.J., Sansom M.S.P. (2011). Molecular simulation approaches to membrane proteins. Structure.

[bib34] Stansfeld P.J., Hopkinson R.J., Ashcroft F.M., Sansom M.S.P. (2009). The PIP_2_ binding site in Kir channels: definition by multi-scale biomolecular simulations. Biochemistry.

[bib35] Tajkhorshid E., Nollert P., Jensen M.O., Miercke L.J.W., O’Connell J., Stroud R.M., Schulten K. (2002). Control of the selectivity of the aquaporin water channel family by global orientational tuning. Science.

[bib36] Tani K., Mitsuma T., Hiroaki Y., Kamegawa A., Nishikawa K., Tanimura Y., Fujiyoshi Y. (2009). Mechanism of aquaporin-4’s fast and highly selective water conduction and proton exclusion. J. Mol. Biol..

[bib37] Ulmschneider M.B., Sansom M.S.P. (2001). Amino acid distributions in integral membrane protein structures. Biochim. Biophys. Acta.

[bib38] van Gunsteren W.F., Kruger P., Billeter S.R., Mark A.E., Eising A.A., Scott W.R.P., Huneberger P.H., Tironi I.G. (1996). Biomolecular Simulation: The GROMOS 96 Manual and User Guide.

[bib39] White S.H. (2009). Biophysical dissection of membrane proteins. Nature.

[bib40] Williamson I.M., Alvis S.J., East J.M., Lee A.G. (2002). Interactions of phospholipids with the potassium channel KcsA. Biophys. J..

[bib41] Yau W.M., Wimley W.C., Gawrisch K., White S.H. (1998). The preference of tryptophan for membrane interfaces. Biochemistry.

