# Supplementary material for: Multiscale Simulations Reveal Conserved Patterns of Lipid Interactions with Aquaporins
Source: Structure. 2013 May 7;21(5):810–9. doi: 10.1016/j.str.2013.03.005 (PMC3746155; doi:10.1016/j.str.2013.03.005)
Supplement: Document S1. Figures S1–S5 and Table S1 [file mmc1.pdf]

Structure, Volume *21*

Supplemental Information

Multiscale Simulations Reveal Conserved Patterns of Lipid Interactions with Aquaporins

Phillip J. Stansfeld, Elizabeth E. Jefferys, and Mark S.P. Sansom

Inventory of Supplemental Information

SI Figure S1, related to Figure 5 - Montage of Aqp structures.

SI Figure S2, related to Figure 2 - Protein-lipid interactions for 3M9I.

SI Figure S3, related to Figure 3 - Lipid density and the thickness of the lipid bilayer around 2B6O.

SI Figure S4, related to Figure 6 - Sequence alignment of all 40 Aqp structures.

SI Figure S5, related to Figure 5 - The mapping of the β-OG detergent from atomistic to CG.

SI Table S1, related to Figure 6 - Aquaporin Structures Investigated.

Movie S1, related to Figure 1 and Figure 8.

Supporting Information for:

Multiscale Simulations Reveal Conserved Patterns of Lipid Interactions with Aquaporins

Phillip J. Stansfeld, Elizabeth E. Jefferys, and Mark S.P. Sansom

SI Figure S1, related to Figure 5 – Montage of Aqp structures (grey ribbon) with bound detergents or lipids (yellow) in the crystal structure.

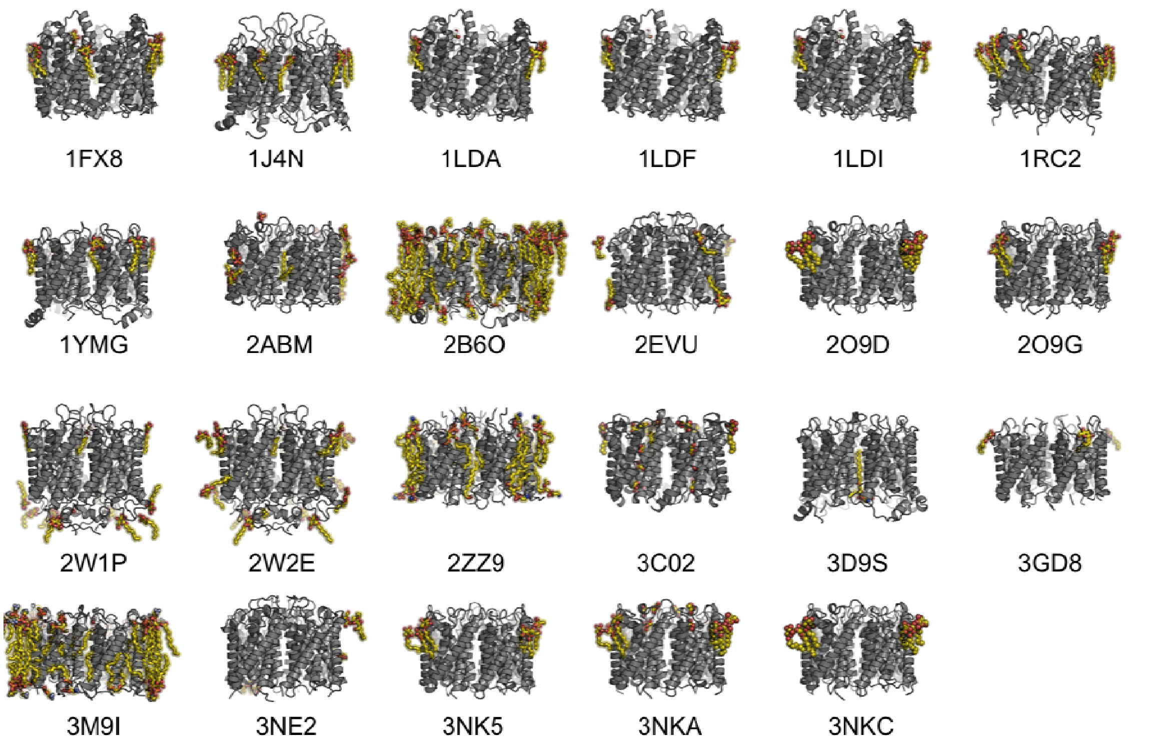

SI Figure S2A, related to Figure 2 Protein-lipid headgroup (red) and acyl tail (green) interactions for 3M9I for the electron crystallographic structure and compared with the contacts made during the CG and atomistic simulations. The contacts are coloured onto their corresponding residues in a white-red gradient for the headgroups or a white-green gradient for the lipid tails.

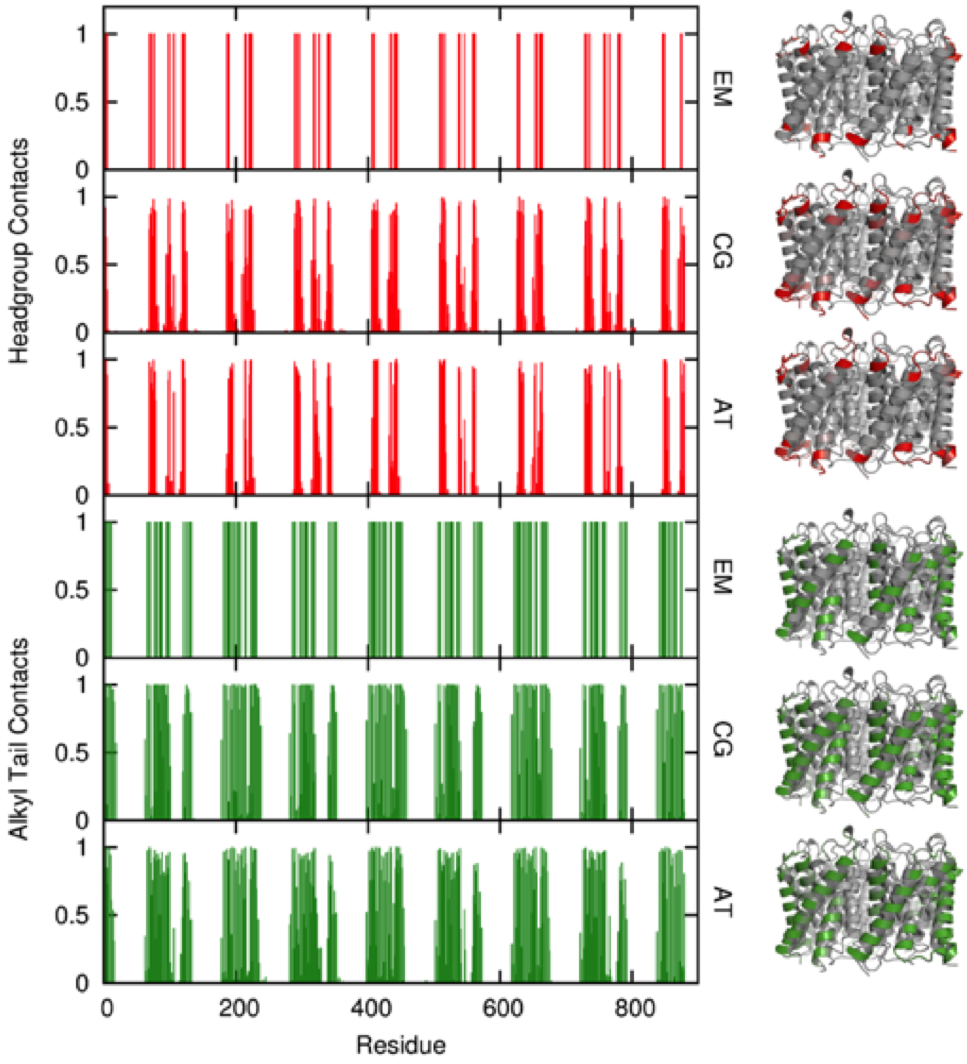

SI Figure S2B, related to Figure 2

Comparison of the contacts for a monomer in complex with lipids. In this case, contacts for the EM system are calculated from atomistic simulations of the EM protein-lipid complex and compared with the CG and AT simulations.

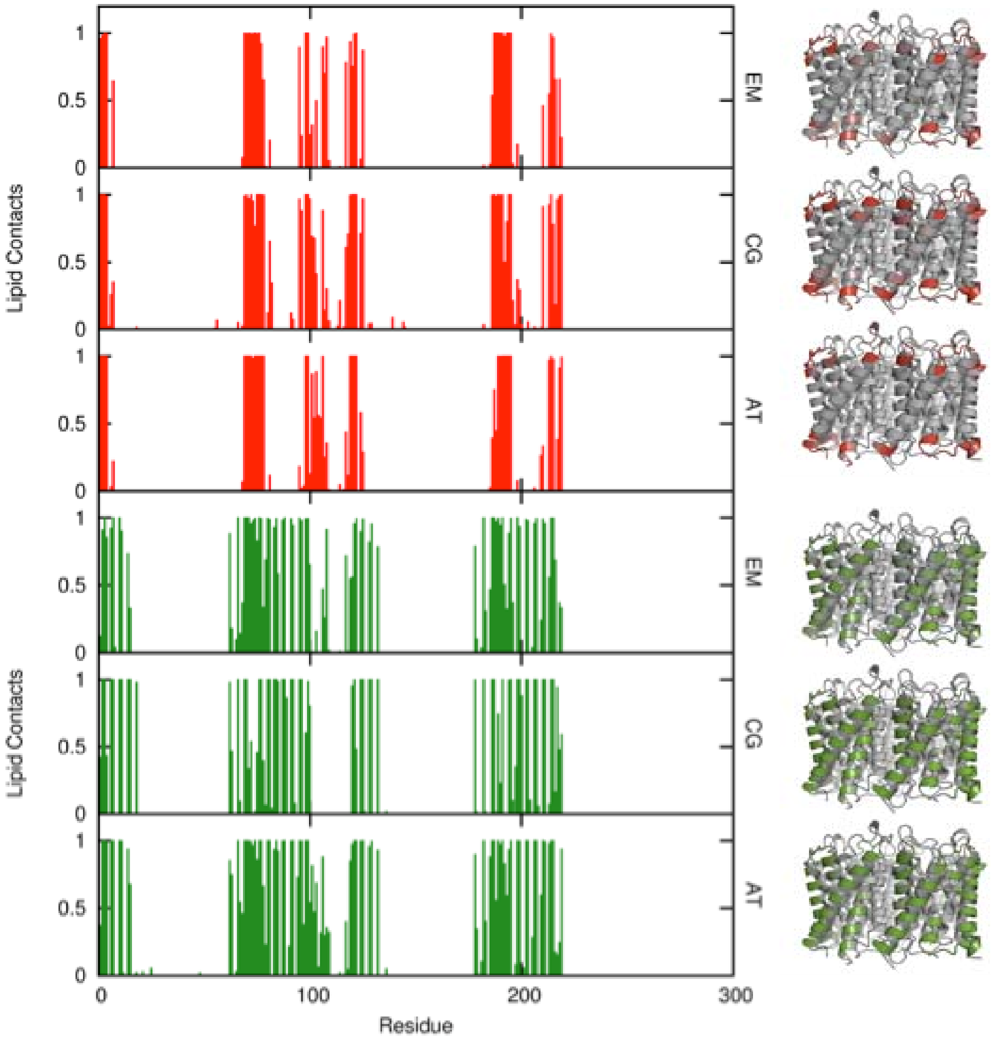

SI Figure S3, related to Figure 3 – (A) Lipid density in the upper (i.e. extracellular) leaflets around 2B6O. The phosphate density is shown on a white to red gradient, with an annulus of lipids clearly apparent. The observed density is compared to the lipids resolved in the electron crystals, with the phosphate atoms of the resolved lipids shown in black spheres. (B) The thickness of the lipid bilayer around 2B6O with DMPC. The thickness of the DMPC bilayer remains at a constant ~35 Å.

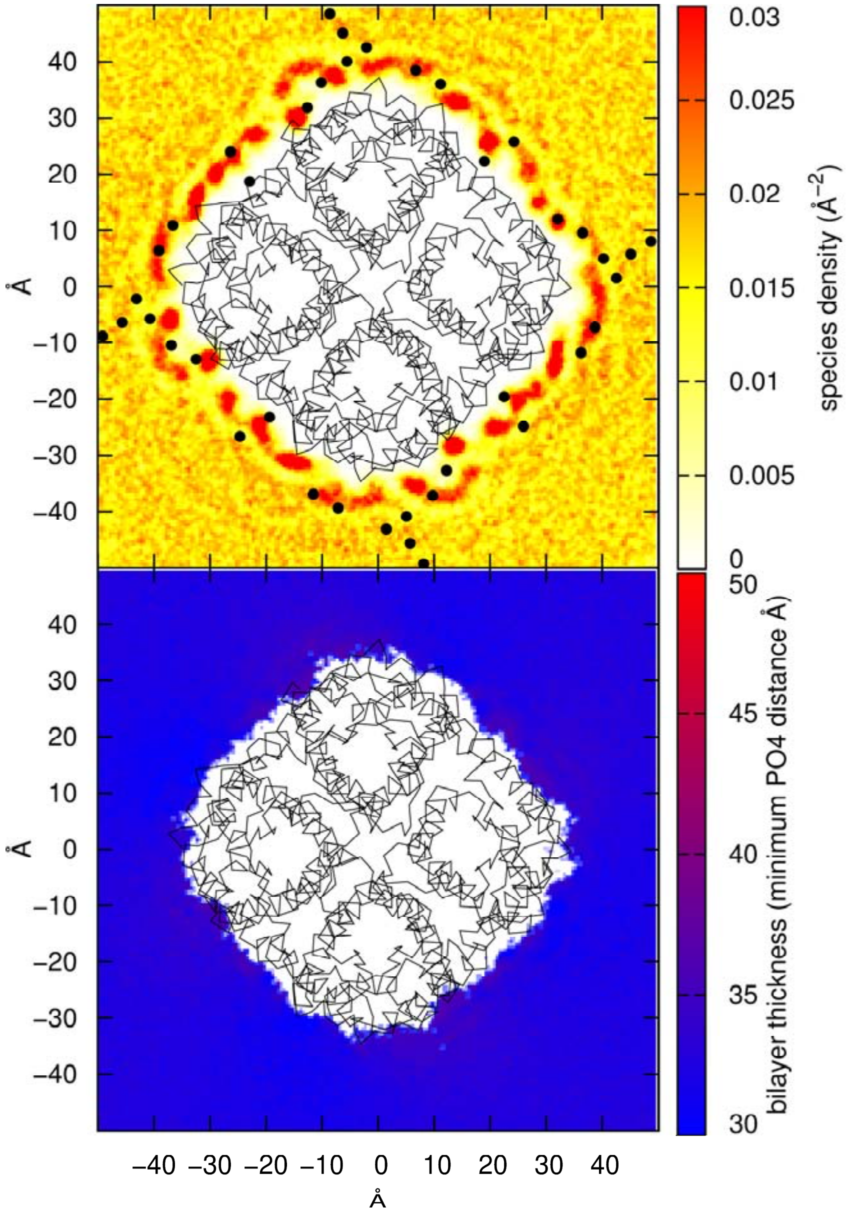

SI Figure S4, related to Figure 6 – Sequence alignment of all 40 Aqp structures. The contacts made by residues with the acyl tails during the simulations are coloured on a green gradient.

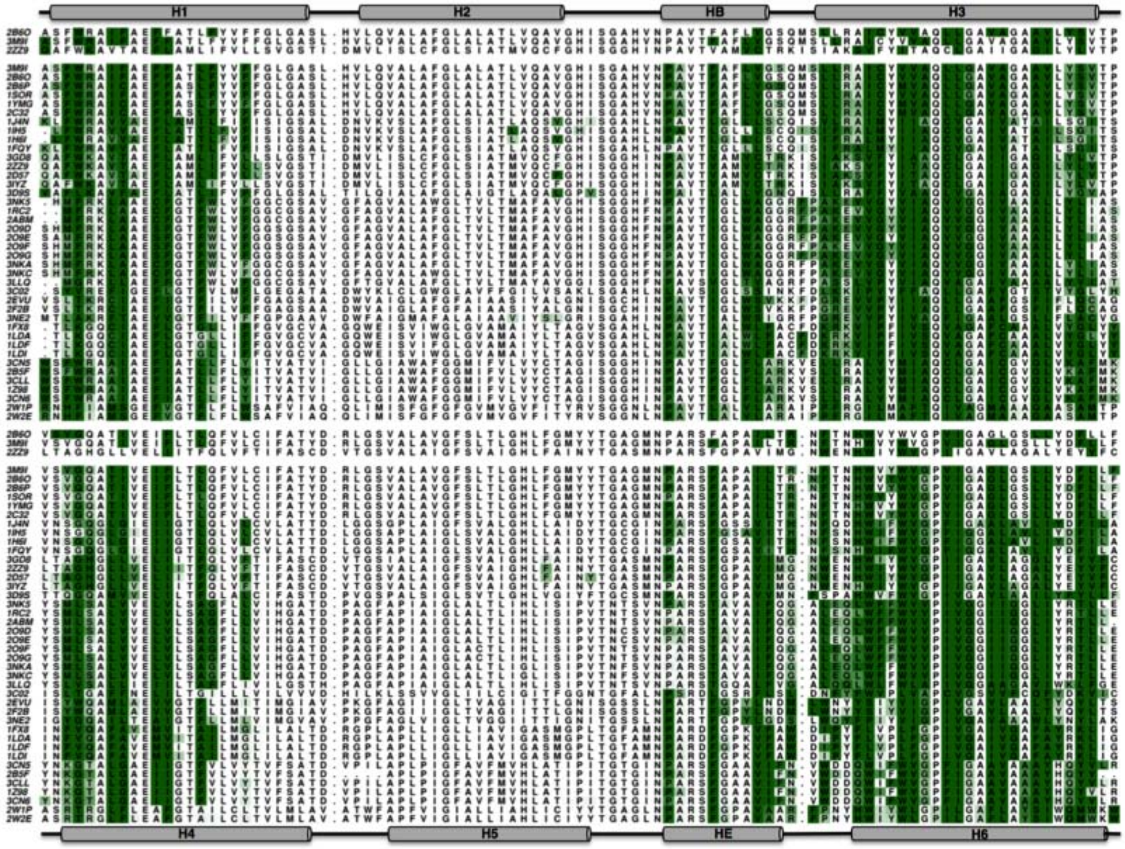

SI Figure S5, related to Figure 5 – The mapping of the β-OG detergent from atomistic to CG. A snapshot from a CG simulation of GlpF in the presence of β-octyl glucoside (β-OG) detergent micelles, indicating how the detergent molecules may act to replicate the membrane environment. The mapping of the β-OG detergent from atomistic to CG is also shown.

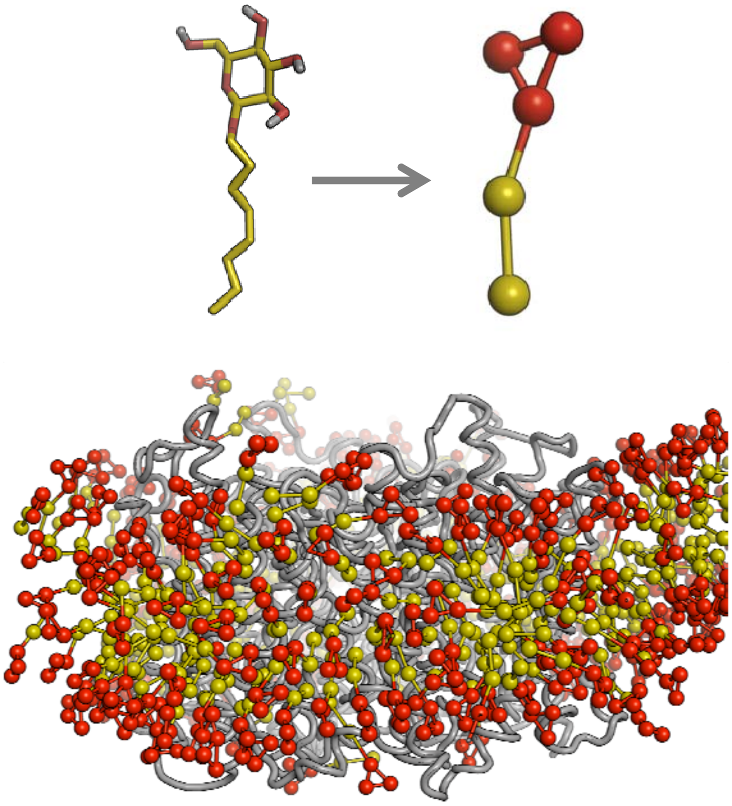

SI Table S1, related to Figure 6 – Aquaporin Structures Investigated

AQP	PDB ID	Species	Exp Method	Resolution	Lipid/Detergent	REF
AQP0	1YMG	*Bos Taurus*	X-ray	2.24	βNG	(1)
AQP0	2C32	*Bos Taurus*	X-ray	7.01	None	(2)
AQP0	1SOR	*Ovis aries*	Electron	3.0/3.5	None	(3)
AQP0	2B6O	*Ovis aries*	Electron	1.90	DMPC	(4)
AQP0	2B6P	*Ovis aries*	Electron	2.40	None	(4)
AQP0	3M9I	*Ovis aries*	Electron	2.50	DSPE	(5)
AQP1	1J4N	*Bos taurus*	X-ray	2.20	βNG	(6)
AQP1	1FQY	*Homo sapiens*	Electron	3.80	None	(7)
AQP1	1H6I	*Homo sapiens*	Electron	-	None	(8)
AQP1	1IH5	*Homo sapiens*	Electron	3.70	None	(9)
AQP4	3GD8	*Homo sapiens*	X-ray	1.80	βOG	(10)
AQP4	2D57	*Rattus norvegicus*	Electron	3.20	None	(11)
AQP4	2ZZ9	*Rattus norvegicus*	Electron	2.80	DSPE	(12)
AQP4	3IYZ	*Rattus norvegicus*	Electron	10.00	None	(13)
AQP5	3D9S	*Homo sapiens*	X-ray	2.00	6-PS	(14)
AqpM	3NE2	*A. fulgidus*	X-ray	3.00	βOG	Lee
AqpM	2EVU	*M. marburgensis*	X-ray	2.30	βOG	(15)
AqpM	2F2B	*M. marburgensis*	X-ray	1.68	None	(15)
AqpZ	3LLQ	*A. tumerfaciens*	X-ray	2.01	None	Liu?
AqpZ	1RC2	*E. coli*	X-ray	2.50	βOG	(16)
AqpZ	2ABM	*E. coli*	X-ray	3.20	DSPE/βOG	(17)
AqpZ	2O9D	*E. coli*	X-ray	2.30	HSG/HSH	(18)
AqpZ	2O9E	*E. coli*	X-ray	2.20	None	(18)
AqpZ	2O9F	*E. coli*	X-ray	2.55	None	(18)
AqpZ	2O9G	*E. coli*	X-ray	1.90	βOG	(18)
AqpZ	3NK5	*E. coli*	X-ray	2.40	βOG	(19)
AqpZ	3NKA	*E. coli*	X-ray	2.50	βOG	(19)
AqpZ	3NKC	*E. coli*	X-ray	3.10	βOG	(19)
GlpF	1FX8	*E. coli*	X-ray	2.20	βOG	(20)
GlpF	1LDA	*E. coli*	X-ray	2.80	βOG	(21)
GlpF	1LDF	*E. coli*	X-ray	2.10	βOG	(21)
GlpF	1LDI	*E. coli*	X-ray	2.70	βOG	(21)
PfAQP	3C02	*P. falciparum*	X-ray	2.05	βOG	(22)
PIP2	1Z98	*S. oleracea*	X-ray	2.10	None	(23)
PIP2	2B5F	*S. oleracea*	X-ray	3.90	None	(23)
PIP2	3CLL	*S. oleracea*	X-ray	2.30	None	(24)
PIP2	3CN5	*S. oleracea*	X-ray	2.05	None	(24)
PIP2	3CN6	*S. oleracea*	X-ray	2.95	None	(24)
Aqy1	2W1P	*P. pastoris*	X-ray	1.40	βOG	(25)
Aqy1	2W2E	*P. pastoris*	X-ray	1.15	βOG	(25)

References

1. Harries WE, et al. (2004) The channel architecture of aquaporin 0 at a 2.2-A resolution *Proceedings of the National Academy of Sciences of the United States of America* 101:14045-50.
2. Palanivelu DV, et al. (2006) Co-axial association of recombinant eye lens aquaporin-0 observed in loosely packed 3D crystals *Journal of Molecular Biology* 355:605-11.
3. Gonen T, et al. (2004) Aquaporin-0 membrane junctions reveal the structure of a closed water pore *Nature* 429:193-7.
4. Gonen T, et al. (2005) Lipid–protein interactions in double-layered two-dimensional AQP0 crystals *Nature* 438:633-638.
5. Hite RK, Li Z, Walz T (2010) Principles of membrane protein interactions with annular lipids deduced from aquaporin-0 2D crystals *EMBO J* 29:1652-1658.
6. Sui H, et al. (2001) Structural basis of water-specific transport through the AQP1 water channel *Nature* 414:872-8.
7. Murata K, et al. (2000) Structural determinants of water permeation through aquaporin-1 *Nature* 407:599-605.
8. de Groot BL, Engel A, Grubmüller H (2001) A refined structure of human aquaporin-1 *FEBS Lett* 504:206-211.
9. Ren G, et al. (2001) Visualization of a water-selective pore by electron crystallography in vitreous ice *Proc Natl Acad Sci USA* 98:1398-1403.
10. Ho JD, et al. (2009) Crystal structure of human aquaporin 4 at 1.8 A and its mechanism of conductance *Proceedings of the National Academy of Sciences of the United States of America* 106:7437-42.
11. Hiroaki Y, et al. (2006) Implications of the aquaporin-4 structure on array formation and cell adhesion *Journal of Molecular Biology* 355:628-39.
12. Tani K, et al. (2009) Mechanism of aquaporin-4's fast and highly selective water conduction and proton exclusion *J Mol Biol* 389:694-706.
13. Mitsuma T, et al. (2010) Influence of the cytoplasmic domains of aquaporin-4 on water conduction and array formation *Journal of Molecular Biology* 402:669-81.
14. Horsefield R, et al. (2008) High-resolution x-ray structure of human aquaporin 5 *Proc Natl Acad Sci USA* 105:13327-32.
15. Lee JK, et al. (2005) Structural basis for conductance by the archaeal aquaporin AqpM at 1.68 A *Proceedings of the National Academy of Sciences of the United States of America* 102:18932-7.
16. Savage DF, et al. (2003) Architecture and selectivity in aquaporins: 2.5 a X-ray structure of aquaporin Z *PLoS Biol* 1:E72.
17. Jiang J, Daniels BV, Fu D (2006) Crystal structure of AqpZ tetramer reveals two distinct Arg-189 conformations associated with water permeation through the narrowest constriction of the water-conducting channel *J Biol Chem* 281:454-60.
18. Savage DF, Stroud RM (2007) Structural basis of aquaporin inhibition by mercury *Journal of Molecular Biology* 368:607-17.
19. Savage DF, et al. (2010) Structural context shapes the aquaporin selectivity filter *Proceedings of the National Academy of Sciences of the United States of America* 107:17164-9.
20. Fu D, et al. (2000) Structure of a glycerol-conducting channel and the basis for its selectivity *Science* 290:481-6.
21. Tajkhorshid E, et al. (2002) Control of the selectivity of the aquaporin water channel family by global orientational tuning *Science* 296:525-30.

22. Newby ZE, et al. (2008) Crystal structure of the aquaglyceroporin PfAQP from the malarial parasite Plasmodium falciparum *Nat Struct Mol Biol* 15**:**619-25.
23. Tornroth-Horsefield S, et al. (2006) Structural mechanism of plant aquaporin gating *Nature* 439**:**688-94.
24. Nyblom M, et al. (2009) Structural and functional analysis of SoPIP2;1 mutants adds insight into plant aquaporin gating *Journal of Molecular Biology* 387**:**653-68.
25. Fischer G, et al. (2009) Crystal Structure of a Yeast Aquaporin at 1.15 angstrom Reveals a Novel Gating Mechanism *Plos Biology* 7**:**13.

Movie S1, related to Figure 1
