# Supplementary figures and images for: Multiscale Simulations Reveal Conserved Patterns of Lipid Interactions with Aquaporins
Source: Structure. 2013 May 7;21(5):810–9. doi: 10.1016/j.str.2013.03.005 (PMC3746155; doi:10.1016/j.str.2013.03.005)

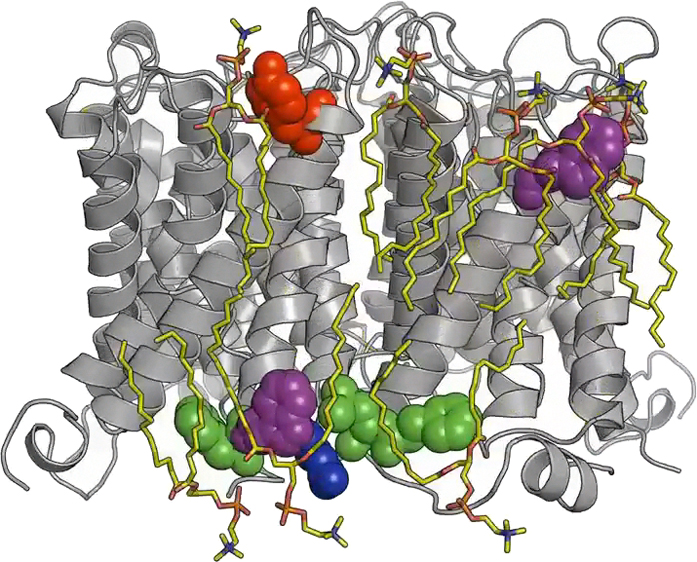

Supplement: Movie S1. Lipid Interactions with Aqp0, Related to Figures 1 and 8 [file mmc2.jpg]
